# Observations on the Ultrastructure of Antennal Sensilla of Adult *Glenea cantor* (Cerambycidae: Lamiinae)

**DOI:** 10.1093/jisesa/ieaa013

**Published:** 2020-03-19

**Authors:** Zishu Dong, Yubin Yang, Fugen Dou, Yujing Zhang, Huixin Huang, Xialin Zheng, Xiaoyun Wang, Wen Lu

**Affiliations:** 1 Guangxi Key Laboratory of Agric-Environment and Agric-Products Safety, College of Agriculture, Guangxi University, Nanning, Peoples R China; 2 Texas A&M AgriLife Research Center, Beaumont, TX

**Keywords:** antennal sensilla, scanning electron microscopy, Cerambycidae, *Glenea cantor*, plant protection

## Abstract

The external morphology and distribution of antennal sensilla of *Glenea cantor* Fabricius were studied with scanning electron microscopy. The antennae of *G. cantor* were observed to be filiform, consisting of scape, pedicel, and flagellum (nine flagellomeres). Four distinct types of sensory receptors were observed, including sensilla chaetica, sensilla trichodea, sensilla basiconica, and Böhm bristles. Three morphological subtypes of sensilla chaetica were found on the antennae, and sensilla trichodea were also categorized into three morphological subtypes. Sensilla basiconica was grouped into two morphological subtypes that were found on subsegments F2-F9 of the flagellum, and Böhm bristles were only found at the intersegmental joints between the scape and the head and between the scape and the pedicel. The antennae of male and female adults were similar in shape, length, and diameter. However, the length, diameter, distribution, and number of each of the four distinct types of sensilla on the males were significantly different from those on females. The types, lengths, diameters, numbers, and distributions of these sensilla were described, and their possible functions were also discussed. The results indicated that the base and end of an antennal segment have a similar sensillum density, but the middle section sensor density is significantly greater, especially for olfactory and gustatory sensilla, possibly because the joints are more involved in mechanical sensing. The density of sensors is closely related to its sensing function; so, future studies on the biology of olfaction and sexual communication in *G. cantor* will be facilitated by these observations.

Cerambycidae is a cosmopolitan family of beetles with more than 36,000 species described around the world (Lu et al. 2007, Wang 2017). As phytophagous insects, Cerambycidae are economically important pests of forests, street trees, and fruits (Dong 2017). Glenea *cantor* Fabricius is a longhorn beetle whose larvae bore under the bark of living trees of at least seven plant families in Southeast Asia, entering the wood for pupation (Lu et al. 2013a). *Glenea cantor* Fabricius is an important pest of *Bombax ceiba* (Linnacus) (Malvales: Bombacaceae) (Lu et al. 2011a, Lu et al. 2013b) and *Adansonia digitata* (Linnaeus) (Malvales: Bombacaceae). The *Bombax ceiba* L. was found to be a new important host plant of the longhorn beetle in Nanning, Guangxi Province, China in 2018. The baobab tree has multiple uses and is highly valued in Southern Africa, particularly in rural communities, where people depend on this resource for their livelihoods (Munyebvu 2018). Information on this new potential invasive pest is therefore critical for more effective control on its damage to *Bombax ceiba* L.

Insect antennae are segmented appendages that are well-equipped. An antenna’s surface is covered by a wide variety of antennal sensilla, which have primary functions of chemoreception and mechanoreception. Chemosensation is of crucial importance to most insects, regulating and triggering various behaviors. Sensilla are specialized structures of the epidermis, especially in the forms of hairs, pegs, and so on (Keil 2012). A mechanoreceptor can allow an insect to experience the degrees of various movements and the size of a host plant (Ma et al. 2019). According to their morphology, the sensilla are termed as Böhm bristles, chaetica, trichoid, basiconica, etc. Sensilla play an important role in the reception of various stimuli (odor, sound, heat, cold, humidity, and tactile information) involved in finding suitable habitats and locating mates (Barbara et al. 2003, Spathe et al. 2013, Ruschioni et al. 2015).

The functions of sensilla have been confirmed by electrophysiological technology and antennal transcriptome analysis (Zhang 2018). Studies of antennal sensilla have mainly been carried out in Diptera, Lepidoptera, and Hymenoptera because of the significant economic value of insects of these orders (Yao et al. 2005, Mark et al. 2017, Wu et al. 2018). In recent years, studies have increasingly focused on antennal sensilla and their ultrastructure in Coleoptera (Chen et al. 2014, Ali et al. 2017), but no such study in *G. cantor* has been conducted. Various options have been explored to control the *G. cantor*, including physical control, chemical control, and biological approaches. For example, several parasites have been found to be effective (Lu 2007). Compared with other control methods, biological control is the most promising way to manage a stem-boring pest over large areas. Semiochemicals are natural chemical compounds that play important roles in the behavioral ecology of insects due to their function in modifying insect behavior. Semiochemicals play an important role as an alternative to traditional methods of pest control, and their development has progressed to reduce the levels of pesticide use.

The purpose of this study was to characterize and determine the antennal morphology and sensillar ultrastructure of the ceiba longhorn beetle *G. cantor* in both sexes using scanning electron microscopy (SEM). This descriptive work will provide a better understanding of the behavioral mechanisms in this stem-boring pest. Coupled with sensory physiology, the results of our study may provide an effective control measure for this pest with semiochemicals.

## Materials and Methods

### Insects

In June 2018, we collected three kapok plants from Qingxiu Mountain (22°12′-23°32′N, 107°45′-108°51′E), Nanning, China, that had withered in the upper stalk due to *G. cantor* infestation and cut them into 10 sections (10–15 cm in diameter × 40 cm in length). The stalk sections were placed in four bubble containers in a yarn cages (40 cm × 80 cm × 80 cm) sprayed with water once every 7 d in a controlled environment (25 ± 1°C, 70 ± 5% RH, 14 L: 10 D; Lu et al. 2011b). After 1.5 mo, newly emerged adults from the cages were randomly collected and transferred to separate glass containers (4 cm in diameter × 12 cm in length). These adults were fed with kapok twigs (2–4 cm in diameter × 8 cm in length), which were renewed every 2 d. We selected five male and five female adults of *G. cantor* and placed them in a freezer at −20°C. After 30 min, the adults were removed and their antennae cut off under a stereomicroscope PX-1 (Camsonar Technology Co., Ltd., Beijing, China). The antennae were stored in a 70% alcohol solution until they were examined.

### Preparation of Specimens

The antennae were cleaned three times in an ultrasonic bath JP-010T (Skymen Cleaning Equipment CO., Ltd., Shenzhen, China) at 250 W for 300 s each. The antennae were then fixed separately in 2.5% glutaraldehyde at 4°C for 12 h. The antennae were dehydrated through an ascending ethanol series of 75%, 80%, 85%, 90%, 95%, and 100% with 10 min intervals. The prepared antennae were stored in a glass container with a desiccant and dried for 24 h. After drying, the samples were mounted on a holder using double-sided sticky tape (dorsal, ventral), sputter coated with gold-palladium, and then observed under a SEM (model S-3400 N, Hitachi, Japan) operated at 5–10 kV. Images were digitally recorded and stored on a computer.

### Data Analysis

**Fig. 1.**
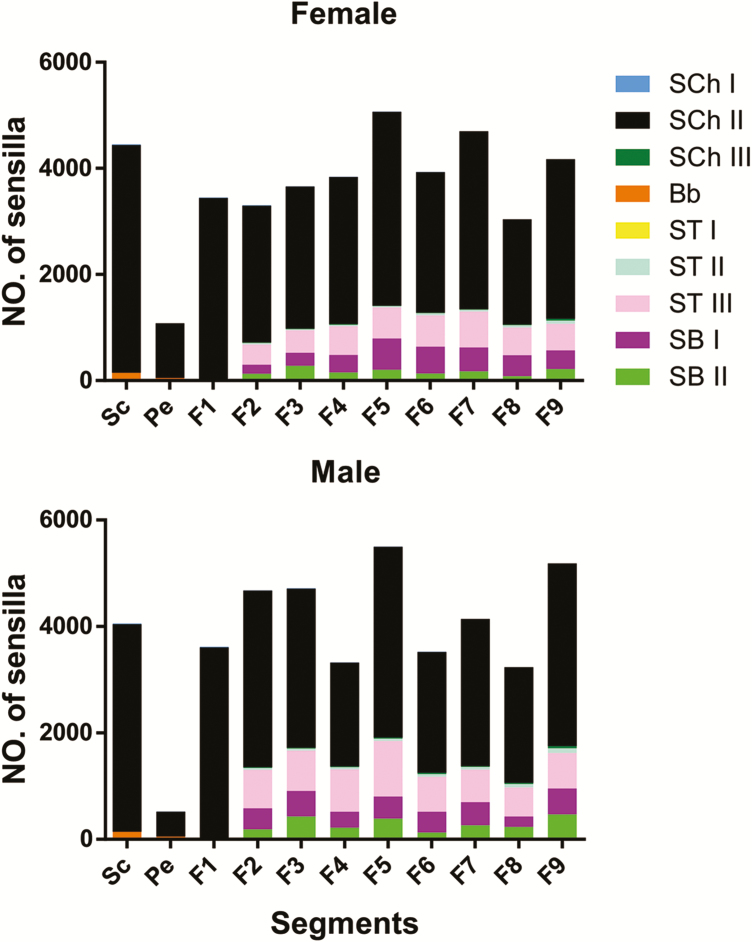
Numbers and distributions of sensilla on the antennae of both sexes of *G. cantor*.

The identification and classification of the sensillum types and the terminology used in this work were based on the studies of Schneider (1964) and Zacharuk (1985). The number of sensilla on the dorsal and ventral surfaces of each antennomere were counted, and the lengths and diameters of sensilla were measured. According to the SEM results, some kinds of sensilla (SChⅡ, STⅢ, SBⅠ, and SBⅡ) with a very high density, small samples were randomly selected at the base, middle, and end of each antennal subsegment (Fig. 1). Each sample was magnified 300 times, and the area of each was approximately 4,444 μm^2^. The number of sensors in each sample was recorded, and the average value was taken. Then, the number of sensors in the area was extrapolated to the corresponding value of the actual area of the site, and the data were statistically analyzed. Sensilla that had relatively low numbers (Bb, SChⅠ, SChⅢ, STⅠ, and STⅡ) were counted individually.

The sensillar distribution patterns were precisely described using Adobe Photoshop software, Version CS6. The distribution of each type of sensillum was analyzed between the dorsal and ventral surfaces of the antennae in both sexes. In all, five individual structures per type were subjected to a quantitative analysis of sensilla. The *t*-test was applied to determine the possible sexual dimorphism of antennal sensilla between males and females using the SPSS statistical software package, version 25.0 (SPSS Inc., Chicago, IL). Student’s *t*-test or analysis of variance were applied to determine the possible differences in of the number, length, and width of antennal sensilla, and the values are reported as means ± standard error (SE). The significance level was set at 0.05.

## Results

### Gross Morphology of Antennae

The numbers of segments were the same for female and male *G. cantor* adults. The antennae of *G. cantor* were filiform, each consisting of a scape, pedicel, and flagellum (nine flagellomeres, F1-F9). The scapes were cylindrically shaped and located in the antennal socket between the compound eyes. Each pedicel was a trapezoid and clearly shorter than the scape and each flagellomere. The nine flagellomeres of each flagellum with blunt tips were similar in shape but different in size. There were no significant differences between males and females in the length or diameter of the antennae (Table 1). The average lengths of scapes and flagellomeres in males were not significantly different from those in females, with an average scape length of 1.75 ± 0.05 mm for males and 1.67 ± 0.13 mm for females (Table 1). Each flagellomere was a barrel-shaped segment with a length of 13.78 ± 0.31 mm and 13.46 ± 0.56 mm for both males and females, respectively (Table 1). The average length (male = 0.39 ± 0.04 mm, female = 0.48 ± 0.03 mm, *t* = −4.40, *P* = 0.02) and diameter (male = 0.33 ± 0.01 mm, female = 0.38 ± 0.01 mm, *t* = −2.76, *P* = 0.03) of pedicels in females were dramatically and significantly greater than those in males (Table 1).

**Table 1.** Lengths and diameters of antennal segments of both sexes in *G. cantor*

Antennal segments	Length(mm)		Statistics		Diameter(mm)		Statistics	
	♀	♂	*t*	*P*	♀	♂	*t*	*P*
Scape	1.67 ± 0.13	1.75 ± 0.05	0.56	0.60	0.55 ± 0.01	0.49 ± 0.03	−1.74	0.14
Pedicel	0.48 ± 0.01*	0.39 ± 0.04	−4.40	0.02	0.38 ± 0.01*	0.33 ± 0.01	−2.76	0.03
Flagellum	13.46 ± 0.56	13.78 ± 0.31	0.49	0.64	0.23 ± 0.01	0.23 ± 0.01	0.30	0.77
Antennae	15.62 ± 0.65	15.91 ± 0.37	0.39	0.70	0.27 ± 0.01	0.26 ± 0.01	−0.42	0.69

Data are presented as means ± SE, *n* = 5.

*indicates significant difference between male and female in length or diameter at 0.05 level using the *t*-test.

The lengths and diameters of the nine flagellomeres were similar in both sexes. There was a significant difference in the diameter of the fourth flagellomere (male = 0.25 ± 0.00 mm, female = 0.24 ± 0.01 mm, *t* = 2.92, *P* = 0.02) between the sexes (Table 2). The nine flagellomeres became gradually shorter and narrower towards the distal ends of the flagellum (Table 2).

**Table 2.** Lengths and widths of flagellomeres of both sexes in *G. cantor*

	sex	Flagellomeres								
		F1	F2	F3	F4	F5	F6	F7	F8	F9
Length (mm)	♀	1.76 ± 0.08a	1.62 ± 0.08ab	1.81 ± 0.08a	1.63 ± 0.07ab	1.60 ± 0.07ab	1.42 ± 0.05bc	1.32 ± 0.05cd	1.12 ± 0.05d	1.19 ± 0.06cd
	♂	1.72 ± 0.08a	1.63 ± 0.02a	1.78 ± 0.03a	1.67 ± 0.04a	1.64 ± 0.03a	1.44 ± 0.03b	1.46 ± 0.07b	1.15 ± 0.05c	1.27 ± 0.02c
Diameter (mm)	♀	0.28 ± 0.01a	0.28 ± 0.01a	0.24 ± 0.01ab	0.24 ± 0.01ab	0.21 ± 0.01bc	0.21 ± 0.01bc	0.20 ± 0.01bc	0.20 ± 0.01bc	0.19 ± 0.01c
	♂	0.27 ± 0.01a	0.28 ± 0.01a	0.24 ± 0.02ab	0.25 ± 0.00ab*	0.23 ± 0.01abc	0.22 ± 0.01abc	0.20 ± 0.02bc	0.19 ± 0.02bc	0.18 ± 0.01c

Data are presented as means ± SE, *n* = 5.

*indicates significant difference between males and females in lengths or diameters at the 0.05 level using the *t*-test. Different letters in each row indicate significant difference at 0.05 level using Tukey’s.

### Types of Antennal Sensilla

**Fig. 2.**
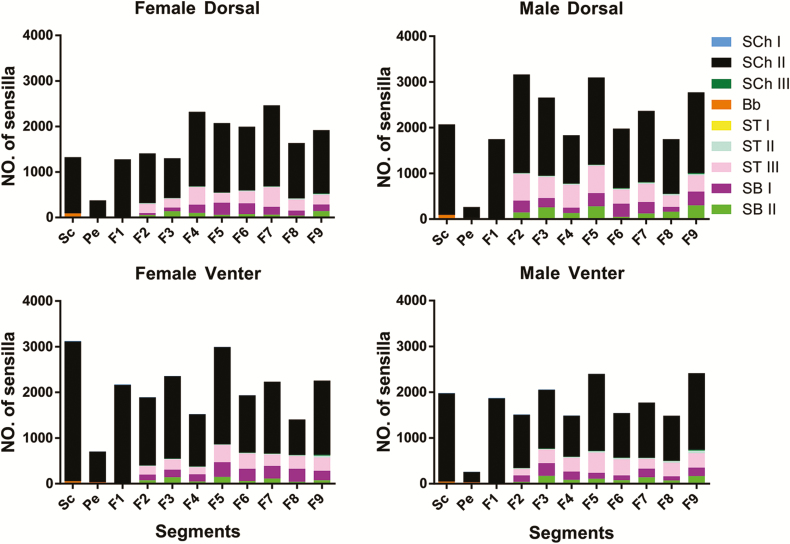
Numbers and distributions of sensilla on the dorsal and venter of the antennae of different segments of both sexes of *G. cantor.*

Nine types of sensilla were identified on the antennae, including Böhm bristles (Bb), sensilla chaetica (SCh I, II, and III), sensilla trichodea (ST I, II, and III), and sensilla basiconica (SB I and II). The numbers and distributions of these sensilla in different segments of both sexes are shown in Fig. 1, and their distribution on the back and venter is shown in Fig. 2. The lengths and widths of these sensilla are shown in Table 3.

**Table 3.** Lengths and diameters of sensilla on the antennae of both sexes in *G. cantor*

Type of sensilla		Sex	Scape	Pedicel	Flagellum								
					F1	F2	F3	F4	F5	F6	F7	F8	F9
SCh I	Length/μm	♀	208.86 ± 21.75	170.43 ± 20.06	191.58 ± 31.39	229.37 ± 21.92	258.03 ± 23.30	256.06 ± 14.67	221.73 ± 10.16	248.53 ± 24.91	—	—	—
		♂	242.85 ± 18.37	221.13 ± 7.31*	249.44 ± 16.42	383.63 ± 24.75*	366.67 ± 29.01*	260.66 ± 16.71	272.83 ± 25.50	295.85 ± 21.68	—	—	—
	Diameter/μm	♀	9.65 ± 1.26	10.07 ± 1.77	9.66 ± 0.86	15.40 ± 2.24	14.86 ± 0.98	11.07 ± 0.43	10.30 ± 0.64	8.67 ± 0.59	—	—	—
		♂	12.36 ± 1.06	12.00 ± 1.13	11.65 ± 1.01	13.10 ± 0.57	13.00 ± 0.70	9.52 ± 0.67	10.18 ± 0.70	8.15 ± 0.40	—	—	—
SCh II	Length/μm	♀	77.86 ± 3.21	62.62 ± 1.92	68.65 ± 2.37	53.25 ± 2.26	57.05 ± 3.10	49.25 ± 3.53	61.80 ± 5.94	43.57 ± 0.87	41.43 ± 1.73	43.50 ± 1.15	50.93 ± 4.82
		♂	76.14 ± 3.41	59.43 ± 2.49	66.35 ± 2.36	53.25 ± 1.30	50.83 ± 0.71	44.67 ± 2.05	50.70 ± 2.59	46.37 ± 1.49	43.57 ± 0.93	44.40 ± 1.90	42.30 ± 1.09
	Diameter/μm	♀	8.29 ± 0.38*	6.88 ± 0.20*	7.35 ± 0.26*	7.05 ± 0.22	7.35 ± 0.35*	6.25 ± 0.25	7.27 ± 0.79	5.40 ± 0.20	4.83 ± 0.19	5.07 ± 0.22	5.90 ± 0.36*
		♂	7.15 ± 0.16	5.64 ± 0.34	6.15 ± 0.17	6.20 ± 0.40	5.63 ± 0.27	5.77 ± 0.35	8.60 ± 1.17	5.74 ± 0.33	4.50 ± 0.09	5.60 ± 0.45	3.92 ± 0.13
SCh III	Length/μm	♀	—	—	167.03 ± 10.18	148.45 ± 7.62	126.55 ± 9.12	132.70 ± 11.41	105.67 ± 9.42	103.94 ± 5.90	110.13 ± 6.87	121.03 ± 7.04	82.13 ± 10.17
		♂	—	—	143.61 ± 4.16	138.04 ± 6.22	120.98 ± 11.83	112.06 ± 3.65	112.11 ± 7.33	105.15 ± 6.73	100.33 ± 5.71	114.16 ± 7.43	67.27 ± 2.28
	Diameter/μm	♀	—	—	9.60 ± 0.40	13.47 ± 1.39*	14.08 ± 0.62*	10.00 ± 0.39	11.35 ± 1.02*	8.31 ± 0.64	6.83 ± 0.47	8.20 ± 0.57	7.20 ± 0.51*
		♂	—	—	9.05 ± 0.13	10.15 ± 0.33	8.43 ± 1.00	9.43 ± 1.11	8.17 ± 0.84	7.90 ± 0.37	6.87 ± 0.43	7.91 ± 0.74	4.83 ± 0.13
Bb	Length/μm	♀	49.21 ± 5.08*	49.17 ± 8.62	—	—	—	—	—	—	—	—	—
		♂	32.21 ± 6.06	42.52 ± 5.29	—	—	—	—	—	—	—	—	—
	Diameter/μm	♀	8.92 ± 0.48*	6.50 ± 0.59*	—	—	—	—	—	—	—	—	—
		♂	7.30 ± 0.33	4.67 ± 0.38	—	—	—	—	—	—	—	—	—
ST I	Length/μm	♀	548.86 ± 16.52*	—	—	—	—	—	—	—	—	—	—
		♂	462.71 ± 20.05	—	—	—	—	—	—	—	—	—	—
	Diameter/μm	♀	11.67 ± 0.75*	—	—	—	—	—	—	—	—	—	—
		♂	8.71 ± 0.34	—	—	—	—	—	—	—	—	—	—
ST II	Length/μm	♀	—	—	53.65 ± 2.91	46.92 ± 2.97	51.65 ± 4.30	45.43 ± 2.37	49.57 ± 1.71	44.00 ± 3.82	50.17 ± 3.55	54.20 ± 4.45	50.20 ± 2.78
		♂	—	—	57.81 ± 4.07	54.00 ± 3.52	55.17 ± 5.95	56.22 ± 3.59*	52.37 ± 3.31	49.02 ± 3.33	53.27 ± 3.64	62.53 ± 3.94	53.11 ± 3.67
	Diameter/μm	♀	—	—	5.54 ± 0.68	4.02 ± 0.19	5.85 ± 0.18	6.48 ± 0.28	4.50 ± 0.09	3.00 ± 0.19	4.07 ± 0.24	4.20 ± 0.09	3.63 ± 0.14
		♂	—	—	6.64 ± 0.53	4.87 ± 0.28*	6.57 ± 0.39	7.67 ± 0.42*	5.83 ± 0.82	4.32 ± 0.20*	4.37 ± 0.17	4.67 ± 0.13*	4.67 ± 0.36*
ST III	Length/μm	♀	—	—	—	23.35 ± 2.77	20.89 ± 1.02	23.40 ± 1.29	24.20 ± 2.00	19.33 ± 0.97	19.33 ± 1.04	22.90 ± 1.21	21.07 ± 1.24
		♂	—	—	—	27.05 ± 1.55	25.10 ± 0.90*	28.50 ± 1.67*	39.27 ± 5.44*	25.30 ± 1.46*	22.13 ± 1.20	25.60 ± 2.12	18.46 ± 1.20
	Diameter/μm	♀	—	—	—	3.15 ± 0.21	3.40 ± 0.26*	2.40 ± 0.22	2.60 ± 0.16	2.77 ± 0.07*	2.63 ± 0.08*	2.17 ± 0.17	2.20 ± 0.23*
		♂	—	—	—	3.20 ± 0.37	1.60 ± 0.16	1.97 ± 0.11	2.35 ± 0.17	1.60 ± 0.07	1.97 ± 0.08	2.00 ± 0.07	1.53 ± 0.13
SB I	Length/μm	♀	—	—	—	15.00 ± 1.09	15.45 ± 1.16*	13.40 ± 1.38	18.80 ± 2.52	14.73 ± 0.72	12.70 ± 1.04	12.63 ± 0.60	11.67 ± 1.37
		♂	—	—	—	12.95 ± 0.89	12.24 ± 0.51	12.35 ± 1.09	18.30 ± 2.53	14.10 ± 1.08	11.97 ± 0.92	11.10 ± 0.81	10.43 ± 0.9a
	Diameter/μm	♀	—	—	—	2.95 ± 0.17*	3.20 ± 0.24*	2.53 ± 0.13*	2.57 ± 0.09*	2.70 ± 0.08*	2.63 ± 0.08*	2.23 ± 0.14*	2.13 ± 0.22*
		♂	—	—	—	2.25 ± 0.17	1.30 ± 0.06	1.92 ± 0.10	1.57 ± 0.13	1.43 ± 0.07	1.87 ± 0.05	1.86 ± 0.10	1.39 ± 0.05
SB II	Length/μm	♀	—	—	—	14.10 ± 0.65	19.21 ± 0.88	12.33 ± 0.42	11.40 ± 0.73	12.67 ± 0.65	11.23 ± 1.21	13.17 ± 0.58	12.03 ± 0.50
		♂	—	—	—	14.99 ± 0.83	19.43 ± 2.49	12.60 ± 0.46	12.75 ± 0.48	13.50 ± 0.33	12.54 ± 0.75	12.90 ± 0.47	12.08 ± 0.56
	Diameter/μm	♀	—	—	—	4.87 ± 0.26*	3.77 ± 0.30*	4.13 ± 0.30*	4.27 ± 0.17*	4.00 ± 0.13*	3.97 ± 0.16	3.03 ± 0.10	2.90 ± 0.21*
		♂	—	—	—	3.61 ± 0.27	2.82 ± 0.19	3.28 ± 0.23	3.40 ± 0.16	3.10 ± 0.19	3.97 ± 0.21	2.93 ± 0.08	2.20 ± 0.20

Data are presented as means ± SE, *n* = 10.

*indicates significant difference between males and females at 0.05 level. ‘—’ indicates the sensilla were absent.

### Böhm Bristles

**Fig. 3.**
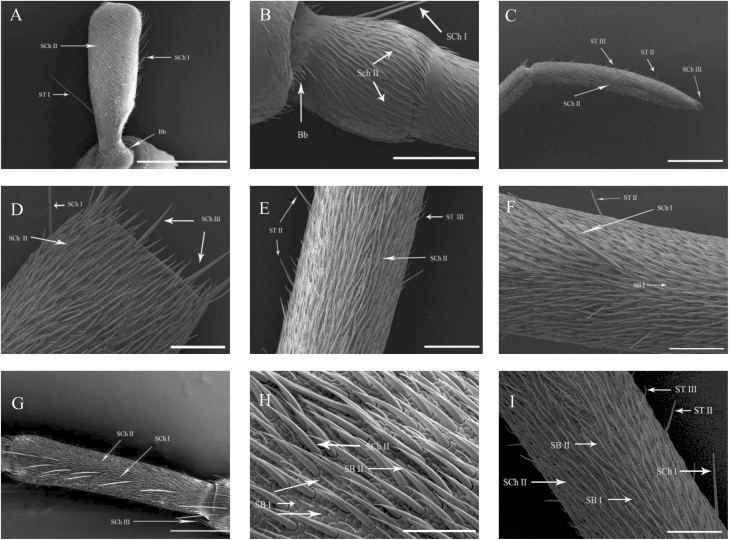
Morphological characteristics of antennae of both sexes of *G. cantor*. (A) Dorsal view of the scape (Sc) of the female. (B) Dorsal view of the pedicel (Pe) of the male. (C) Ventral view of the ninth flagellum of the male. (D) Dorsal view of the fifth flagellum of the female. (E) Dorsal view of the seventh flagellum of the female. (F) Ventral view of the fifth flagellum of the female. (G) Ventral view of the second flagellum of the female. (H) Dorsal view of the third flagellum of the male. (I) Ventral view of the fifth flagellum of the male. Scale bars: (A) = 1,000 μm; (C, G) = 500 μm; (B)= 300 μm; (D, E, F), (I) = 100 μm; (H) = 50 μm.

**Fig. 4.**
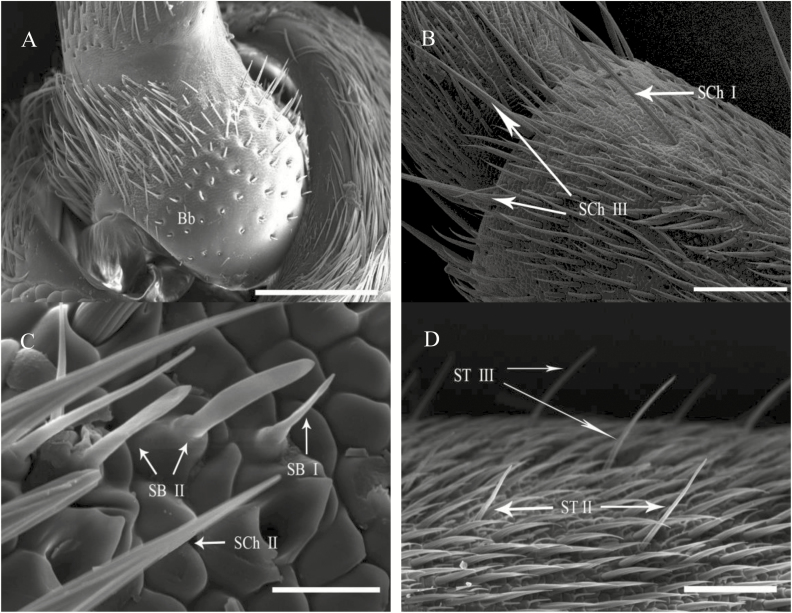
High-resolution images for types of sensillum on the antennae of both sexes of *G. cantor.* (A) Dorsal view of the scape (Sc) of the female. (B) Ventral view of the fourth flagellum of the male. (C) Back view of the fifth flagellum of the female. (D) Dorsal view of the sixth flagellum of the male. Scale bars: (A) = 200 μm; (B) = 100 μm; (D)= 50 μm; (C) = 10 μm.

Böhm bristles are a special type of sensillum chaeticum located only on the joint region between the ommateum and the scape and on the joint region between the scape and the pedicel. The lengths and diameters of Böhm’s bristles (Bb) were greater in females than in males. The average length of Bb on Sc in females (49.21 ± 5.08 μm) was dramatically and significantly longer than that in males (32.21 ± 6.06 μm), but not significantly different between females (49.17 ± 8.62 μm) and males (42.52 ± 5.29 μm; Table 3) on pedicel. The average diameter of Bb on scape and pedicel in females (scape: 8.92 ± 0.48 μm, pedicel: 6.50 ± 0.59 μm) was dramatically and significantly greater than those in males (scape: 7.30 ± 0.33 μm, pedicel: 4.67 ± 0.38 μm; Table 3). The cuticle walls had shallow grooves and were nonporous (Fig. 3A and B, Fig. 4A).

### Sensilla Chaetica

Sensilla chaetica are widely distributed on antennae. These sensilla are more robust than other mechanical sensilla. These sensilla could be further classified into three subtypes based on length and shape: SCh I, II, and III.

- Sensilla chaetica type I (SCh I) are clearly recognizable, and their tips protrude beyond all other sensilla. These sensilla are at nearly a 45° angle to the surface of the antenna (Fig. 3A,B, and I) and are long, robust, and slightly curved hairs with strong longitudinal grooves and a blunt tips. However, SCh I is located only on the ventral side of scape, pedicel, and some joints of flagellomeres: F1-F6 (F1 = the first flagellomere, Table 3, Fig. 3G). The average length of SCh I showed significant differences between females and males on pedicel (male = 221.13 ± 7.31 μm, female = 170.43 ± 20.06 μm), the second flagellomere (male =383.63 ± 24.75 μm, female = 229.37 ± 21.92 μm), the third flagellomere (male = 336.67 ± 37.78 μm, female = 258.03 ± 23.30 μm). The average diameter of SCh I, however, did not differ significantly between females and males.
- Sensilla chaetica type II (SCh II) are the most numerous antennal sensilla; they are studded on all joints of antennae on each side. They are robust, groove-walled, and stout hairs. The diameter of this subtype of sensillum slowly increases distally, then suddenly decreases near the end of the sensillum to form a pointed tip (Fig. 3A–E and G–I, Fig. 4C). Their average length did not differ significantly between females and males. The average diameter was significantly different between females and males on scape (male = 7.15 ± 0.16 μm, female = 8.29 ± 0.38 μm), pedicel (male = 5.64 ± 0.34 μm, female = 6.88 ± 0.20 μm), the first flagellomere (male = 6.15 ± 0.17 μm, female = 7.35 ± 0.26 μm), the third flagellomere (male = 5.63 ± 0.27 μm, female = 7.35 ± 0.35 μm), and the ninth flagellomere (male = 3.92 ± 0.13 μm, female = 5.90 ± 0.36 μm).
- Sensilla chaetica type III (SCh III) are visible at the junction of each segment of the flagellomeres. These sensilla are long, spiny, robust, groove-walled, and curved with wide sockets; the tips are not porous (Figs. 3D and 4B). Their average length did not differ significantly between females and males. The average diameter differed significantly between females and males on the second flagellomere (male = 10.15 ± 0.33 μm, female = 13.47 ± 1.39 μm), the third flagellomere (male =8.43 ± 1.00 μm, female = 14.08 ± 0.62 μm), the fifth flagellomere (male = 8.17 ± 0.84 μm, female = 11.35 ± 1.02 μm), and the ninth flagellomere (male = 4.83 ± 0.13 μm, female = 7.20 ± 0.51 μm; Table 3).

### Sensilla Trichodea

Sensilla trichodea are hair-like protruding receptors with longitudinal grooves (stripes) or smooth and perforated surfaces, and are widely distributed on the antennae. The base of each sensillum is embedded in a wide or narrow receptor nest, and the morphological structure of sensilla trichodea can be classified into three distinct types: ST I, II, and III.

- Sensilla trichodea type I (ST I) insert into a broad sockets; they are long, slender, straight, or curved with smooth surfaces (Fig. 3A). ST I is present only on the scapes of antennae. ST I were larger in females than in males in length (male = 462.71 ± 20.05 μm, female = 548.86 ± 16.52 μm) and diameter (male = 8.71 ± 0.34 μm, female = 11.67 ± 0.75 μm).
- Sensilla trichodea type II (ST II) are curved hairs with blunt tips. The hair base inserts tightly into a small cuticular socket at a 45–60° angle to the surface of the antenna. Furthermore, ST II are visible at the junctions of flagellomeres (Fig. 3C,E,F, and I, Fig. 4D) and their average length differed significantly between females and males on the fourth flagellomere (male = 56.22 ± 3.59 μm, female = 45.43 ± 2.37 μm). The average diameter showed significant differences between females and males on the second flagellomere (male = 4.87 ± 0.28 μm, female = 4.02 ± 0.19 μm), the fourth flagellomere (male = 7.67 ± 0.42 μm, female = 6.48 ± 0.28 μm), the sixth flagellomere (male = 3.00 ± 0.19 μm, female = 4.32 ± 0.20 μm), the eighth flagellomere (male = 4.20 ± 0.09 μm, female = 4.67 ± 0.13 μm), and the ninth flagellomere (male = 4.67 ± 0.36 μm, female = 3.63 ± 0.14 μm; Table 3).
- Sensilla trichodea type III (ST III) are slightly curved hairs with smooth surfaces and pores (Fig. 3C,E, and I, Fig. 4D). These sensilla possess thin and sharp tips, and are curved at the middle part of the sensilla that inserts into a tight socket. These sensilla are visible at most junctions of flagellomeres (F2-F9, F9 = the ninth flagellomere). The average length differed significantly between females and males on the third flagellomere (male = 25.10 ± 0.90 μm, female = 20.89 ± 1.02 μm), the fourth flagellomere (male = 28.50 ± 1.67 μm, female = 23.40 ± 1.29 μm), the fifth flagellomere (male = 39.27 ± 5.44 μm, female = 24.20 ± 2.00 μm), and the sixth flagellomere (male = 25.30 ± 1.46 μm, female = 19.33 ± 0.97 μm). The average diameter showed significant differences between females and males in the third flagellomere (male = 1.60 ± 0.16 μm, female = 3.40 ± 0.26 μm), the fourth flagellomere (male = 1.97 ± 0.11 μm, female = 2.40 ± 0.22 μm), the sixth flagellomere (male = 1.60 ± 0.07 μm, female = 2.77 ± 0.07 μm), the seventh flagellomere (male = 1.97 ± 0.08 μm, female = 2.63 ± 0.08 μm), and the ninth flagellomere (male = 1.53 ± 0.13 μm, female = 2.20 ± 0.23 μm; Table 3).

### Sensilla Basiconica

Sensilla basiconica are interspersed among the flagellomeres. Generally, a sensillum basiconica has a pedestal shape or a conical uplift base. In the center of the base, there are small cone-shaped receptors of different shapes, which have chemical sensory functions such as smell perception and taste perception. The basiconic sensilla can be further classified into two types based on their surface micro-morphology: Sensilla SB I and II. These sensilla are visible at most junctions of flagellomeres (F2-F9, F9 = the ninth flagellomere).

- Sensilla basiconica I (SB I) are cone-shaped and straight with slightly pointed tips (Fig. 3F,H, and I, Fig. 4C). These sensilla occur on the second to the ninth flagellomere in both sexes. The average diameter in females was significantly greater than in males on F2-F9 (F9 = the ninth flagellomere), but the average length was significantly different only on the third flagellomere (male = 12.24 ± 0.51 μm, female = 15.45 ± 1.16 μm; Table 3).
- Sensilla basiconica II (SB II) are thumb-like shapes, each with a smooth wall and blunt tip. These sensilla are distributed from the second to the ninth flagellomere in both sexes (Fig. 3H and I, Fig. 4C). The average diameter showed differed significantly between females and males on F2-F6 and F9 (F9 = the ninth flagellomere), but the average length was significantly different only on the seventh flagellomere (male = 12.54 ± 0.75 μm, female = 11.23 ± 1.21 μm; Table 3).

## Discussion

**Fig. 5.**
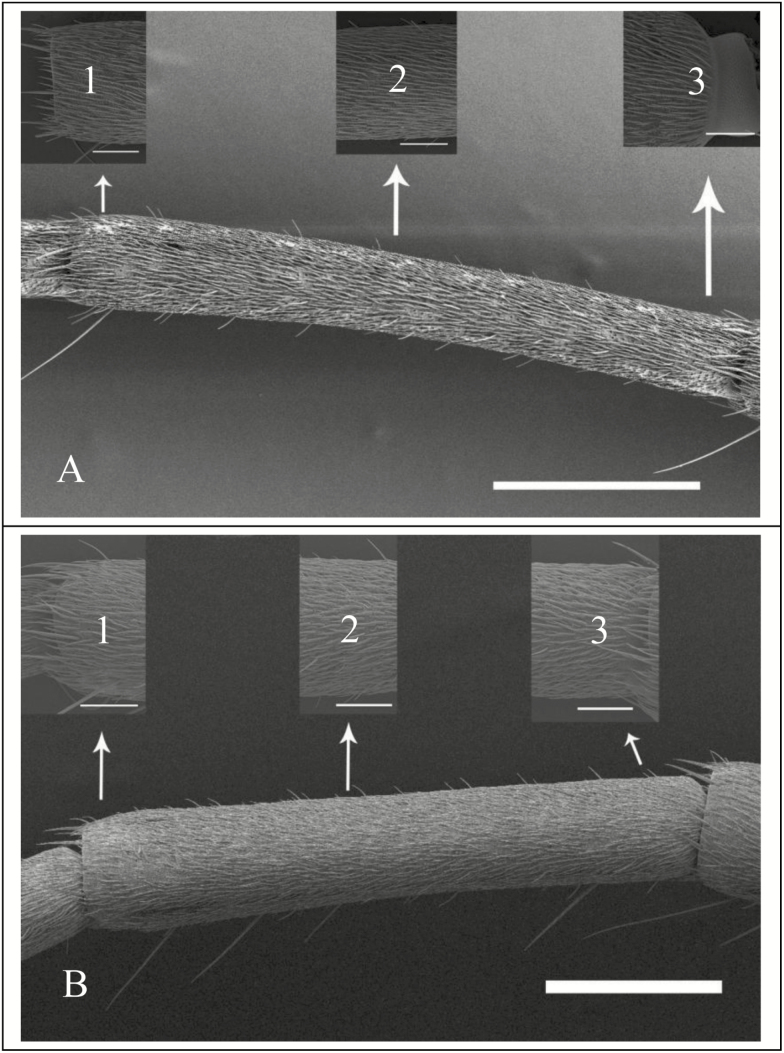
Description of the distribution of antennal sensilla among segments of both sexes of G. cantor. (A) Dorsal view of the fifth flagellum of the female. (B) Dorsal view of the fifth flagellum of the male. (1) Terminal view of the fifth flagellum of the antenna. (2) Middle view of the fifth flagellum of the antenna. (3) Basal view of the fifth flagellum of the antenna. Scale bars: (A, B) = 500 μm; (1, 2, 3) = 100 μm.

The general shapes, structures, and numbers of sensilla in *G. cantor* are largely consistent with those reported for other long-horned beetle species, such as *Tetropium fuscum* (Fabricius) (Coleoptera: Cerambycidae) (Mackay et al. 2014), *Xylotrechus grayii* (White) (Coleoptera: Cerambycidae) (Chen et al. 2014), and *Monochamus alternatus* (Hope) (Coleoptera: Cerambycidae) (Ali et al. 2017). In this study, random samples were taken from the base, middle, and end of each antennal subsegment of antenna due to differences in the density and distribution at different joints. The results indicated that for olfactory and gustatory sensilla (ST and SB), the base and end of an antennal segment show a similar sensilla density, but the middle section exhibits a significantly greater sensor density. Mechanical sensilla (SCh and Bb), however, are widely distributed in the joints and abdomen (Fig. 5), possibly because the joints are more involved in mechanical sensing. Previous studies have also shown that the density of sensors is closely related to sensing function (Gill et al. 2013, Liu et al. 2013).

The antennae of *G. cantor* are similar in size, shape, and structure in both sexes, which is consistent with previous research (Lu 2007). There were no significant differences between males and females in the average length or diameter of antennae. However, females have longer bodies (male = 12.99 ± 1.02, female =14.16 ± 0.91, *t* = 9.62, *P <* 0.05), so males have more developed antennae. The results of SEM results of the antennae of the longicorn beetles show that the types of antennal sensilla of females and males are the same, but there are differences in their numbers and distribution. According to their external shape, dimensions, and location, three types of chaeticum, three types of trichodeum, two types of basiconicum, and one type of Böhm bristle were distinguished in both male and female *G. cantor*.

Previous research suggests that SCh and Bb may function as proprioceptors, sensing antennal movement (Chi et al. 2009, Sun et al. 2011). As in many insects, the concentration of Bb at the intersegmental joints between the scape and the head as well as between the scape and the pedicel, indicates that these sensilla probably perceive antennal position and movement (Sun et al. 2011). The results showed that males have more developed sensilla on the back than females (Fig. 2). Additionally, females have more developed venteral sensilla than males (Fig. 2). Males need highly developed sensilla on their back to facilitate searching for mating. Meanwhile, females need well-developed sensilla on their venter for oviposition. As sensilla are located only on the ventral side of scapes, pedicels, and some joints of flagellomeres (F1-F6, F1 = the first flagellomere), the more advanced development of SCh I in male *G. cantor* may increase its mechanosensory perception when climbing on a female’s back, while highly developed SCh II and SCh III in female *G. cantor* may facilitate oviposition.

On the other hand, a role in olfaction and gustation is suggested for ST and SB (Finch et al. 2000, Hallem et al. 2006). In *G. cantor*, the numbers, lengths, and diameters of ST II and ST III on male antennae were greater than on female antennae, probably because males need greater chemoreceptivity to the sex pheromone in addition to the host volatiles. This research shows that males have more robust ST II and ST III than females (Table 3). Moreover, males have more abundant SB I and SB II than females (Fig. 1). In addition, ST I are present only on the venter of the antenna, ST II are visible at the junctions of flagellomeres, and ST III and SB are visible at most joints of flagellomeres (F2-F9, F9 = the ninth flagellomere). Previous studies (Lu 2007) indicate that after the removal of F9 of males for 24 h, the percentages of grasping, bellybending, and successful mating of the male are significantly reduced in males, even though no significant difference was found in the percentage of short-term mating between the treatment and control groups. When F8 and F9 were removed, there were no significant differences in the percentage of holding, curving, or mating between the males and females. When five segments of the flagellomeres (F5-F9, F9 = the ninth flagellomere) of the males were removed, the percentage of grasping, curving, and mating were significantly lower than when only the last one or two terminal segments were removed. When the antennal flagellum of males was removed, males retained 4% of the recorded holding behavior, but there was no further abdominal curvature reaction. When the whole antenna was removed, males no longer showed any mating behavior. The results from the study described above (Lu 2007) and this study confirms that ST and SB act as sex pheromone detectors for *G. cantor*. ST and SB are used by females for host recognition and oviposition.

In summary, this study was designed to characterize the external morphology of antennal sensillum types of *G. cantor* using a SEM, which provided valuable insights into the possible functions of each sensillum. We also evaluated the sexual differences in the number and distribution of various sensilla. These results will help us better understand the host selection and courtship behavior of *G. cantor* as well as help correlate these behaviors with electrophysiological mechanisms in future studies. The observed differences in the sensillar distribution and function will greatly facilitate designing more effective semiochemical control methods for insect pests.
